# Cerebral Pressure Passivity in Newborns with Encephalopathy Undergoing Therapeutic Hypothermia

**DOI:** 10.3389/fnhum.2014.00266

**Published:** 2014-04-24

**Authors:** Rathinaswamy Bhavanandhan Govindan, An N. Massaro, Nickie N. Andescavage, Taeun Chang, Adré du Plessis

**Affiliations:** ^1^Division of Fetal and Transitional Medicine, Children’s National Medical Center, Washington, DC, USA; ^2^Division of Neonatology, Children’s National Medical Center, Washington, DC, USA; ^3^Department of Neurology, Children’s National Medical Center, Washington, DC, USA

**Keywords:** cerebral oximetry, cerebral oxygen extraction, NIRS, spectral analysis, cerebral pressure autoregulation, neonatal encephalopathy, therapeutic hypothermia

## Abstract

We extended our recent modification of the power spectral estimation approach to quantify spectral coherence. We tested both the standard and the modified approaches on simulated data, which showed that the modified approach was highly specific and sensitive to the coupling introduced in the simulation while the standard approach lacked these features. We also applied the modified and standard approaches to quantify the pressure passivity in 4 infants receiving therapeutic hypothermia. This was done by measuring the coupling between continuous cerebral hemoglobin differences and mean arterial blood pressure. Our results showed that the modified approach identified a lower pressure passivity index (PPI, percent time the coherence was above a predefined threshold) than the standard approach (*P* = 0.0027).

## 1 Introduction

Cerebral pressure autoregulation buffers the changes in systemic mean arterial pressure (MAP) in order to regulate cerebral blood flow (CBF); in contrast its failure, cerebral pressure passivity (CPP), results in broad changes in CBF that track changes in MAP (Panerai et al., 1995). In normal subjects, CPP operates in a time frame of 5–20 s (0.05–0.25 Hz) and hence the frequency domain approach, namely spectral coherence is used to characterize CPP (Panerai et al., 1995, 1996; Panerai, 1998, 2008; Tsuji et al., 2000; O’Leary et al., 2009). Two continuous physiological signals needed to quantify CPP are MAP and CBF. Studies based on animal models have shown the hemoglobin difference, HbD (oxygenated hemoglobin minus de-oxygenated hemoglobin) acquired using near infrared spectroscopy (NIRS) is a reliable surrogate for CBF (Soul et al., 1998; Tsuji et al., 1998; O’Leary et al., 2009). To date, NIRS is the only device that provides a non-invasive continuous long-term measurement of changes in CBF at the bedside.

Spectral coherence is a technique that allows quantification of the relationship between changes in MAP and changes in CBF (or HbD). However, the spectral coherence approach is highly sensitive to the non-stationarities in the signals. Recently, a modification has been proposed to the power spectral estimation approach to mitigate the effect of non-stationarity in physiological signals. The modified approach taken in Govindan et al. (2013) is directly extended in the current work to quantify the spectral coherence. The performance of the standard coherence approach and the modified approach is discussed using simulated signals. Additionally, these approaches are applied to quantify CPP in newborns receiving hypothermia treatment for neonatal encephalopathy.

## 2 Materials and Methods

### 2.1 Standard coherence estimation

We used 10 min of HbD and MAP data to quantify CPP using the following steps (Halliday et al., 1995): Step 1: we partitioned the MAP and HbD data into disjointed time windows of 30 s. Step 2: for the data in each window, we calculated periodograms as the square of the magnitude of the Fourier transform of the signals and the cross-spectrum between the signals as the product of the Fourier transform of one signal and the complex conjugate of the Fourier transform of the second signal. Step 3: we averaged the periodograms and the cross-spectra over all the windows to get the estimate of the spectral quantities. We defined coherence as the ratio of the square of the magnitude of the estimate of the cross-spectrum to the product of the estimates of the power spectra of the two signals.

### 2.2 Modified approach

We modified the coherence estimation approach by dividing the data in 30 s epochs from each signal obtained in step 1 (as in the standard coherence estimation) by their standard deviations. Non-stationarities in the data would cause each epoch to have a different variance; as a result, the spectral estimates for non-stationary epochs would be a poor representation of the corresponding quantities; the normalization by the standard deviation would mitigate this spurious variability caused by the non-stationarity and allow a reasonable estimate of the coherence.

The confidence of the coherence estimate at 100α% is given by 1−(1 − α)^1/(*M* − 1)^, where *M* is the number of 30 s epochs involved in the estimation of the coherence. To reliably quantify the coupling between HbD and MAP in the very low-frequency band (0.05–0.25 Hz), a band that might show spurious coherence due to the contribution from very slow trends such as baseline changes in the signals, we set α to 0.999. For this value of α, the formula yielded a confidence limit of 0.384 for the coherence estimate for a 10 min window. Any value of coherence above this confidence limit was considered statistically significant.

### 2.3 Numerical simulation to validate the coherence approaches

We generated Gaussian distributed random numbers of the same number of sample size as the 10 min of HbD and MAP signals (6000) to validate the standard and the modified coherence approaches. We denote this time series as *x*(*t*). We simulated two different scenarios: one to test the sensitivity and another to test the specificity of the two approaches. Since we expected the coupling between MAP and HbD to occur in the low-frequency band of 0.05–0.25 Hz, we bandpass filtered the noise [*x*(*t*)] in the same band using 4th order Butterworth filter with zero-phase distortion. In scenario one, we modified the values of the filtered data at two different time instances as follows: between samples 1001 and 1500, we multiplied the filtered data by a factor of 400. In another instance we multiplied the values of the filtered data between samples 3001 and 4000 by a factor of 200. We denote the modified filtered signal as *y*(*t*). We calculated coherence between *x*(*t*) and *y*(*t*) using the standard and modified approaches to determine which method better captured the coherence between signals.

In scenario 2, we generated an independent random series of 6000 samples. We modified the samples 1001–1500 in this series by multiplying them by a factor of 400. We denote this time series as *z*(*t*). This scenario would mimic simultaneous spurious changes in both signals [*y*(*t*) and *z*(*t*)] not related to physiology. We calculated the coherence between the *z*(*t*) and *y*(*t*) generated in scenario 1 using both methods to determine which method better identified the spurious change.

We simulated 1000 realizations for each scenario and calculated coherence using the standard and modified approaches. For comparison, we considered the maximum coherence in the frequency band of 0.05–0.25 Hz from both approaches.

### 2.4 Clinical data

Continuous recordings of cerebral oximetry (NIRO 200, Hamamatsu Photonics, Hamamatsu, Japan) (HbO_2_, Hb, total oxygenation index), blood pressure from an indwelling arterial line (Philips IntelliVue MP70, MA, USA), arterial oximetry (Masimo Corporation, CA, USA) from four newborns were collected in a time-locked manner at a rate of 1 kHz using a custom software developed in LabView (National Instruments, TX, USA). The newborns were receiving therapeutic hypothermia for encephalopathy according to the National Institute of Child Health and Human Development Protocol (Shankaran et al., 2005). Cerebral oximetry was obtained bilaterally, one from the left and another from the right fronto-temporal regions. We calculated HbD as HbO_2_–Hb for each hemisphere. We calculated MAP for each cycle of the continuous blood pressure signal using a combination of the lowpass filtering and a peak detection approach. We resampled the MAP into uniformly sampled data using cubic spline at a sample rate of 10 Hz. We also down sampled the HbD signal to 10 Hz. All processing was done off-line using MATLAB (Mathworks, Inc). The study was approved by the Children’s National Medical Center Institutional Review Board and informed consent was obtained from the parents of the patients.

### 2.5 Analysis of MAP and HbD

We partitioned the MAP and HbD data into non-overlapping windows of 10 min duration. In each window, we calculated the coherence between MAP and HbD using both the standard and modified approaches. To compare the performance of the each approach, we calculated the pressure passivity index (PPI) as the percent time the data displayed significant coherence during the entire period. We compared the PPI obtained by the modified and standard approaches using the paired *t*-test. A value of *P* < 0.05 was considered statistically significant.

## 3 Results

### 3.1 Simulation results

**Figure 1:**
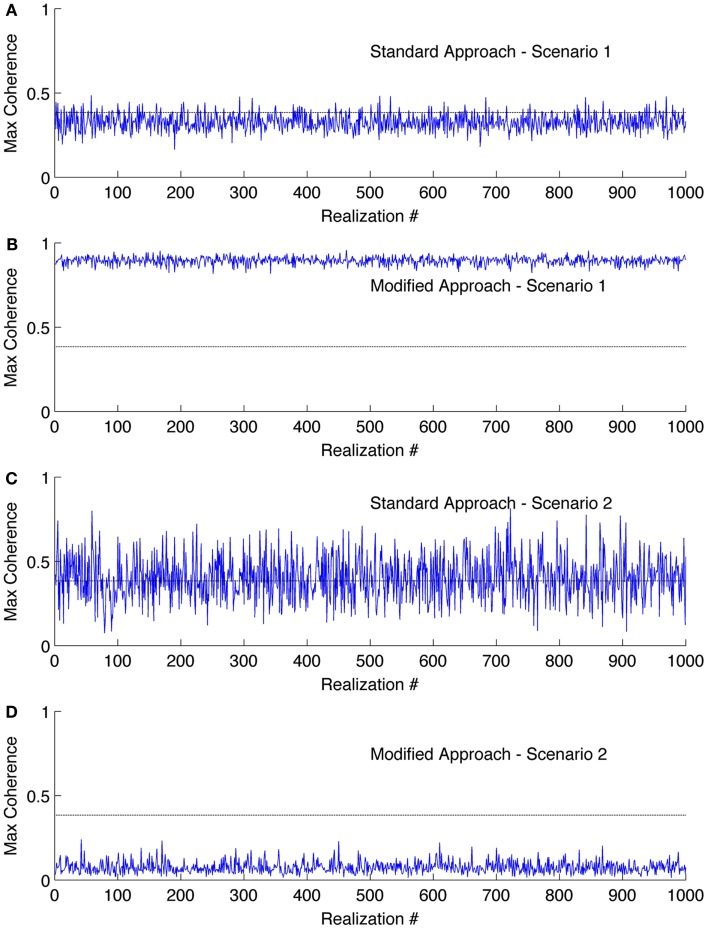
**Results of the coherence analysis of simulated data**. Maximum coherence obtained in 0.05–0.25 Hz for scenario 1 using **(A)** standard approach and **(B)** the modified approach. Coherence obtained in 0.05–0.25 Hz for scenario 2 using **(C)** standard approach and **(D)** the modified approach. The horizontal line at the coherence value of 0.384 is the confidence limit for coherence estimate.

We displayed the results obtained from the simulation study in Figure 1. The standard approach displayed (Figure 1A) maximum coherence in the frequency band of 0.05–0.25 Hz only for 14% of the realizations for scenario 1; in contrary, the modified approach showed coherence in the same frequency band for all the realizations (see Figure 1B). The standard approach showed coherence in the 0.05–0.25 Hz band for about 52% of the realizations for scenario 2 while the modified approach showed coherence in none of the realizations (see Figures 1C,D). Note in scenario 1, we had incorporated coupling in all the realizations whereas in scenario 2 we did not incorporate coupling in any of the realizations.

### 3.2 Clinical results

We studied four newborns receiving therapeutic hypothermia for neonatal encephalopathy. Study monitoring was initiated within 10–20 h after birth. Infants underwent hypothermia treatment for 72 h, followed by gradual rewarming by 0.5°C/h over 6 h to normothermia. Two infants had favorable outcomes (survived with normal MRI) and the other two had adverse outcomes (death, *n* = 1 or severe deep nuclear gray matter injury on MRI, *n* = 1).

### 3.3 Coherence analysis of MAP and HbD signals

**Figure 2:**
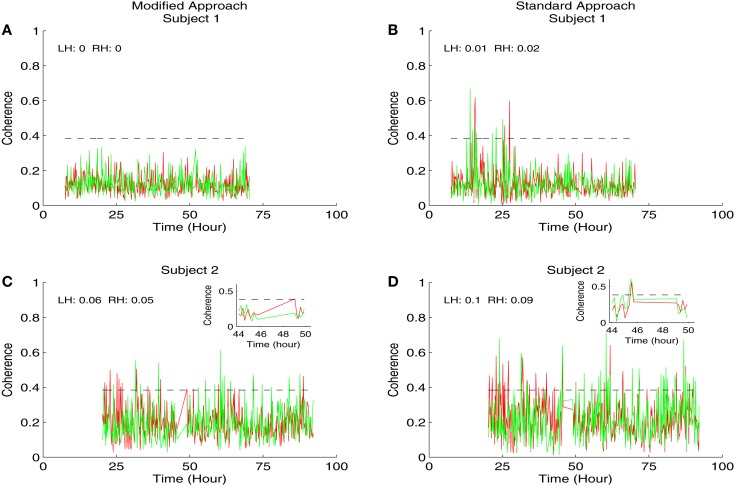
**Results of the coherence analysis of MAP and HbD from two newborns from favorable outcome group receiving hypothermia therapy for neonatal encephalopathy**. The maximum coherence in 0.05–0.25 Hz was shown in all the plots from time since birth. Results from the modified approach were shown in the left side **(A,C)** and the results from the standard approach were shown in the right side **(B,D)**. Results from subject 1 were shown in **(A,B)** and the results from subject 2 were shown in **(C,D)**. In all the plots, the results from the left hemisphere (LH) were shown in red and the results from the right hemisphere (RH) were shown in green. Also the pressure passivity index (PPI) calculated for LH and RH was given in the inset. The horizontal line at the coherence value of 0.384 is the confidence limit for the coherence estimate. We displayed the insets in **(C,D)** for the portion of the results from the period where the study was stopped at 45.5 h and restarted at 49 h. At the onset of the break, the modified approach showed no significant coherence while the standard approach showed a significant coherence in both hemispheres. Similarly at 49 h after the study was restarted, the modified approach showed coherence only in the left hemisphere while at this point the standard approach showed no significant coherence.

**Figure 3:**
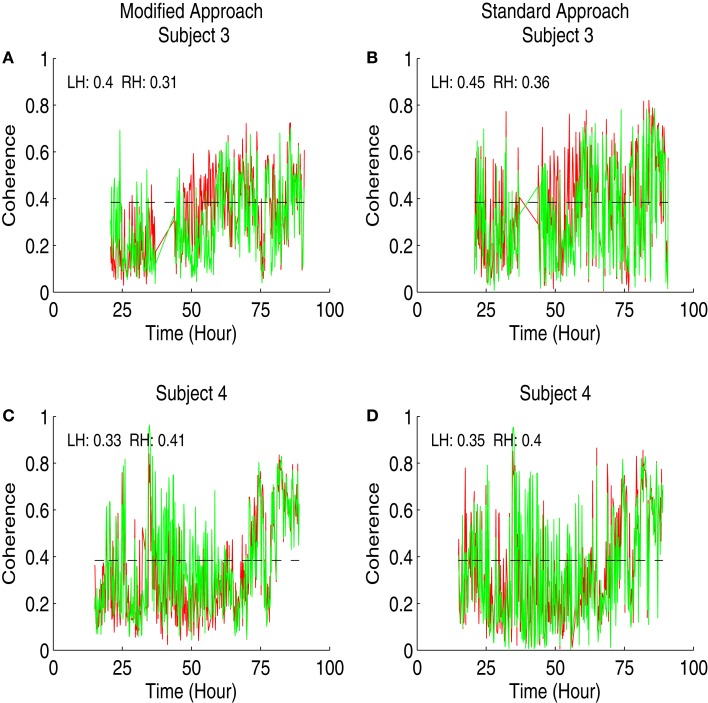
**Results of the coherence analysis of MAP and HbD from two newborns from adverse outcome group receiving hypothermia therapy for neonatal encephalopathy**. The maximum coherence in 0.05–0.25 Hz was shown in all the plots from time since birth. Results from the modified approach were shown in the left side **(A,C)** and the results from the standard approach were shown in the right side **(B,D)**. Results from subject 3 were shown in **(A,B)** and the results from subject 4 were shown in **(C,D)**. In all the plots, the results from the left hemisphere (LH) were shown in red and the results from the right hemisphere (RH) were shown in green. Also the pressure passivity index (PPI) calculated for LH and RH was given in the inset. The horizontal line at the coherence value of 0.384 is the confidence limit for the coherence estimate.

We displayed the results of coherence analysis of the MAP and HbD signals from two newborns that had favorable outcome in Figure 2 and from newborns that had adverse outcome in Figure 3. We showed the maximum coherence between MAP and HbD in 0.05–0.25 Hz. From both approaches, the two infants that had favorable outcomes showed lower PPI values compared to the infants that had adverse outcomes (*P* < 0.001) (see insets in Figures 2 and 3). Further, the modified approach showed lower PPI values compared to the standard approach in most cases. For instance, subjects 2 and 3, had break in the recording between 45.5 and 49 h and 35 and 40 h, respectively. During these instances, the modified coherence did not detect any coupling between HbD and MAP. In contrast, the standard approach falsely detected coupling between HbD and MAP at the edges corresponding to the onset and end of the record breaks where there were no data (see Figures 2C and 3B, wherein coherence from both hemispheres exceeded the confidence limits around the break points). For example, in subject 2, the modified approach detected coherence only after the study was restarted at around 49 h, but only in the left hemisphere (see inset in Figure 2C) while the standard approach missed to detect this association (see inset in Figure 2D). Further, the standard approach detected coherence from both hemispheres when the recording was stopped at 45.5 h (see Figure 2D) but the modified approach did not show any coherence at this time point (see Figure 2C). We did not have enough number of PPI values to run the *t*-test independently for each hemisphere and assess the performance of the approaches. Hence, for each approach we combined the PPI values calculated for the left and right hemispheres and compared them using paired *t*-test. We found a significant difference (*P* = 0.0027) between the results obtained from both approaches with lower PPI values from the modified approach.

## 4 Discussion

We extend our previous work on the modified spectral estimation approach (Govindan et al., 2013) to coherence estimation. Using numerical simulation we demonstrate that the modified approach has high sensitivity and specificity in characterizing the coherence between two signals. We discuss the application of this approach to characterize PPI (a marker of cerebral pressure passivity) as a significant coupling between MAP and HbD.

Scenario 1 in our simulation mimics a surge in the signals that interrupts the detection of coupling between MAP and HbD. On the other hand, scenario 2 mimics a surge in the signals, which falsely characterizes the intact cerebral pressure autoregulation (absence of significant coupling between MAP and HbD) as pressure passivity. The surges introduced in our scenarios are designed to simulate changes in the signals caused by critical care events, for example, endotracheal tube suctioning (Limperopoulos et al., 2008). Such changes may have contributions from physiology, which in turn may affect the ongoing cerebral hemodynamics (cerebral autoregulation). Hence, the methods employed should be robust enough to avoid spurious quantification of CPP. Our numerical simulation results show that the standard approach to estimate coherence lacks sensitivity and specificity in quantifying the association between the two signals during physiologic changes resulting in signal surges. In contrast, the modified approach proposed in this work is highly sensitive and specific in quantifying the association between the signals.

Earlier studies have shown that in critically ill infants, the CPP is not a continuous phenomenon but rather fluctuates in time (Soul et al., 2007; Howlett et al., 2013). Further, infants displaying high PPI values are more likely to have significant cerebrovascular lesions. Thus, it is important to have a reliable method for monitoring CPP in high-risk patients being cared for in the intensive care unit. Perinatal hypoxic ischemic encephalopathy is a major cause of death and disability in children. Our preliminary results show that impaired autoregulation (higher PPI values) can be measured and quantified in HIE newborns who died or had significant MRI injury. In contrast, surviving newborns without MRI injury were observed to have low PPI. Further work is needed, and ongoing, to determine the evolution and importance of CPP in the pathogenesis of perinatal brain injury.

Cerebral pressure passivity identifies the brain unprotected against unstable systemic hemodynamic and oxygenation support. We hypothesize that a sequence of events occurs between the onset of a brain insult and the eventual development of irreversible brain injury. Broadly, this sequence consists of (1) failure of hemodynamic regulation, (2) functional failure (electrocortical dysfunction) followed by (3) failure of the oxygen metabolism as an immediate precursor to permanent structural damage. Failure of hemodynamic regulation is characterized by the PPI analysis. The functional (electrocortical) failure can be studied by measuring the brain activity using SQUID based magnetoencephalographic techniques (Paetau, 2002; Roberts et al., 2014). MEG offers better spatial resolution compared to its electrical homolog electroencephalogram (EEG) (Paetau, 2002). Though both MEG and EEG have submillisecond temporal resolution, MEG has shown to identify deep sources with finer resolution compared to EEG (Paetau, 2002). EEG technology has also evolved over time trending to offer simultaneous dense array measurements from over 100 cortical sites (Welch et al., 2014). Future work would focus on studying simultaneous functional changes of the brain along with NIRS measurements.

## 5 Conclusion

We have demonstrated limitations in the standard coherence approach in quantifying association between two non-stationary signals and propose a modified coherence estimation approach. The modified approach reliably quantifies the association between the signals in the numerical simulations. We will apply the modified coherence estimation approach to a larger patient population to study the relation between PPI and neurologic injury as detected by MRI.

## Conflict of Interest Statement

The authors declare that the research was conducted in the absence of any commercial or financial relationships that could be construed as a potential conflict of interest.
